# Localization of the *Drosophila* Rad9 Protein to the Nuclear Membrane Is Regulated by the C-Terminal Region and Is Affected in the Meiotic Checkpoint

**DOI:** 10.1371/journal.pone.0038010

**Published:** 2012-05-29

**Authors:** Rotem Kadir, Anna Bakhrat, Ronit Tokarsky, Uri Abdu

**Affiliations:** Department of Life Sciences, Ben-Gurion University of the Negev, Beer-Sheva, Israel; Tulane University Health Sciences Center, United States of America

## Abstract

Rad9, Rad1, and Hus1 (9-1-1) are part of the DNA integrity checkpoint control system. It was shown previously that the C-terminal end of the human Rad9 protein, which contains a nuclear localization sequence (NLS) nearby, is critical for the nuclear transport of Rad1 and Hus1. In this study, we show that in *Drosophila*, Hus1 is found in the cytoplasm, Rad1 is found throughout the entire cell and that Rad9 (DmRad9) is a nuclear protein. More specifically, DmRad9 exists in two alternatively spliced forms, DmRad9A and DmRad9B, where DmRad9B is localized at the cell nucleus, and DmRad9A is found on the nuclear membrane both in *Drosophila* tissues and also when expressed in mammalian cells. Whereas both alternatively spliced forms of DmRad9 contain a common NLS near the C terminus, the 32 C-terminal residues of DmRad9A, specific to this alternative splice form, are required for targeting the protein to the nuclear membrane. We further show that activation of a meiotic checkpoint by a DNA repair gene defect but not defects in the anchoring of meiotic chromosomes to the oocyte nuclear envelope upon ectopic expression of non-phosphorylatable Barrier to Autointegration Factor (BAF) dramatically affects DmRad9A localization. Thus, by studying the localization pattern of DmRad9, our study reveals that the DmRad9A C-terminal region targets the protein to the nuclear membrane, where it might play a role in response to the activation of the meiotic checkpoint.

## Introduction

The 9-1-1 complex, comprising the Rad9, Hus1 and Rad1 proteins, is thought to act as part of a DNA damage checkpoint pathway. In response to genotoxic damage, the 9-1-1 complex is loaded onto DNA by a Rad17-containing clamp loader. The DNA-bound 9-1-1 complex then facilitates ataxia telangiectasia-related kinase (ATR) -mediated phosphorylation and activation of Chk1, a protein kinase that regulates S-phase progression, G2/M arrest, and replication fork stabilization. Recent studies have revealed that 9-1-1 proteins physically and functionally interact with key components involved in base excision repair (BER) [1]-[3]. Studies in yeast revealed the role of the 9-1-1 complex in error-prone and error-free post-replication repair (PRR) [4]–[5]. In addition, the *Saccharomyces cerevisiae* 9-1-1 complex was found to be involved in double-strand break (DSB) repair via homologous recombination (HR) [6]–[8]. The 9-1-1 complex was also found to be involved in programed cell death [9]–[11], cell cycle arrest [12] and in both mitotic and meiotic checkpoint responses [1], [13].

The crystal structure of the 9-1-1 complex has been determined shows that 9-1-1 proteins share high structural resemblance to the proliferating cell nuclear antigen [PCNA], despite low sequence identity (14%), as was predicted by earlier bioinformatics analysis [14]. A comparison of each 9-1-1 subunit to PCNA revealed that Rad1 shares the highest structural resemblance to each monomer of PCNA. It was also found that the major differences between the two complexes are assigned to the inter-domain connecting (IDC) loop [15]–[17].

Previous studies in human cell lines revealed that human Rad9 (hRad9) contains a nuclear localization signal (NLS) near the C-terminus of the protein and that this NLS is essential for hRad9 localization to the nucleus. Furthermore, co-expression of hRad9 with either hRad1 or hHus1 resulted in the nuclear localization of these otherwise cytoplasmic proteins, indicating the importance of the NLS in nuclear localization of the human 9-1-1 complex [18]. It was also found that human Rad1 (hRad1) but not hRad9 stabilizes the expression of human Hus1 (hHus1) *in vitro* and acts as a chaperone, stabilizing hHus1 in the cytoplasm [19]. hHus1 was found to be degraded by the ubiquitin-proteasome pathway, with such degradation being suppressed by hRad1 but not by hRad9 [19].

Focusing our initial analysis on the *hus1* gene, we have begun to investigate the function of the 9-1-1 complex in *Drosophila* [20]–[21]. Mutations in *Drosophila hus1* (*DmHus1*) lead to female sterility, suggesting that *DmHus1* plays a role in the meiotic program. *DmHus1* mutation suppresses the dorsal-ventral patterning defects caused by mutations in DNA repair enzymes, suggesting a role for *hus1* in regulating the meiotic DNA damage checkpoint. We also demonstrated that *DmHus1* is required for homologous recombination repair during meiosis [21]. In mitotic cells, we determined that *DmHus1*-mutant flies are sensitive to hydroxyurea and methyl methanesulfonate but not to X-ray irradiation, suggesting that *DmHus1* is required for the activation of an S-phase checkpoint. On the other hand, *DmHus1* is not required for the G2-M checkpoint or for post-irradiation induction of apoptosis [20].

In this study, we addressed the localization pattern of the *Drosophila* 9-1-1 complex and analyzed the importance of the localization pattern of DmRad9A during activation of the meiotic checkpoint.

## Results

### *Drosophila* Rad9A protein is localized to the nuclear membrane in Schneider cells (S_2_R+) and in ovarian follicle cells

**Figure 1:**
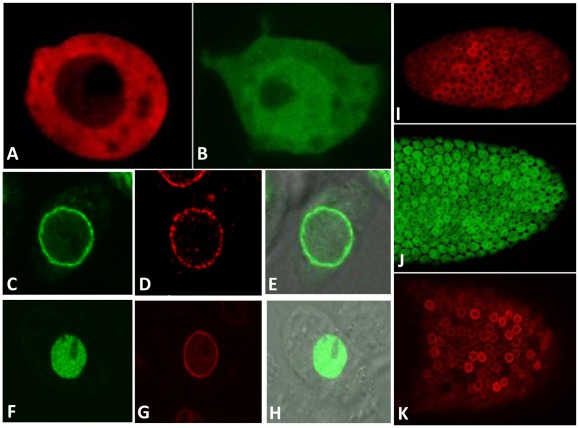
Localization of the *Drosophila* Rad9, Hus1 and Rad1 proteins in S_2_R+ and follicle cells. A–G, Confocal images of S_2_R+ cells, I–K, Confocal images of follicle cells from egg chambers. (A) S_2_R+ cells expressi ng HA-DmHus1 and stained with anti-HA antibodies in red. (B) S_2_R+ cells expressing GFP-DmRad1. (C) S_2_R+ cells expressing GFP-DmRad9A. (F) S_2_R+ cells expressing DmRad9B-GFP. (D) and (G) Staining with anti-lamin antibodies, which mark the nuclear membrane, in red. (E and H) are merged image of (C with differential interference contrast (DIC) image) and (F with a DIC image), respectively. (I) Egg chamber from HA-DmHus1::*CY2Gal4* transgenic flies. (J) Egg chamber from GFP-DmRad1::*CY2Gal4* transgenic flies. (K) Egg chamber from FLAG-DmRad9A::*CY2Gal4* transgenic flies. In both S_2_R+ and follicle cells, DmHus1 is found in the cytoplasm, DmRad1 is found throughout the cell and Dm DmRad9A is localized to the nuclear membrane. DmRad9B is localized to the nucleus in S_2_R+ cells.

To better understand the function of the 9-1-1 complex during *Drosophila* development and in DNA damage checkpoint responses, we analyzed the localization pattern of each of the proteins alone. First, polyclonal antibodies against DmRad9 were raised, however, these antibodies did not work for both western blot and for immunolocalization. Next, a construct in which the endogenous DmRad9 gene was tagged with GFP was generated; however, the tagged protein could not be detected in S_2_R+ or S2 cells. Thus, an alternative approach for studying the localization pattern of the *Drosophila* 9-1-1 complex was chosen. Since previous studies on the localization pattern of the human 9-1-1 complex relied on the expression of tagged protein in mammalian cells [18]–[19], the same approach was adopted here. Thus, tagged versions of each protein that could be expressed in S_2_R+ or S2 cells (*Drosophila*-embryo derived cells) or in transgenic flies using the UAS/Gal4 binary system were created. All of the proteins used in this study were tagged at their N-terminus (see Material and Methods) and were over-expressed using actin-Gal4 promoter. Expression of HA-tagged DmHus1 in S_2_R+ cells and showed that the protein is evenly distributed throughout the cytoplasm (Figure 1A). We also found that GFP-tagged DmRad1 was localized throughout the entire S_2_R+ cell (Figure 1B). Based on the *Drosophila* genome annotation in flybase, it was suggested that the *Drosophila rad9* gene transcribed two alternative splicing forms, *DmRad9A* and *DmRad9B*. Expression of a GFP-tagged versions of these two alternatively spliced variants showed that whereas DmRad9B is concentrated in the S_2_R+ nucleus (Figure 1F), DmRad9A is localized to the S_2_R+ nuclear membrane (Figure 1C), as revealed by co-localization with lamin (Figure 1D, G), a structural component of the nuclear membrane.

To analyze the localization pattern of the 9-1-1 complex in flies, transgenic insects in which a tagged form of each protein could be expressed using the UAS/Gal4 system were created. These genes were cloned into the pUASp vector, which allows for expression in somatic cells, as well as in germline-derived tissues. We first over-expressed the protein in the ovarian somatic follicle cells, since it could be easily detected in these cells, It was found that DmHus1 is localized to the cytoplasm (Figure 1I), that DmRad1 is evenly distributed in the follicle cells (Figure 1J) and that DmRad9A is found at the follicle cell nuclear membrane (Figure 1K).

### The *DmRad9A* transcript is more abundant than is the *DmRad9B* transcript during oogenesis

Our preliminary results showed differences in the localization pattern of the two alternatively spliced DmRad9 proteins. Based on flybase, *DmRad9A* and *DmRad9B* transcripts encode for proteins containing 456 amino acids each. These two alternatively spliced forms share the same first 424 amino acids, with the last 32 amino acids differing between the two DmRad9 forms. Interestingly, *Drosophila* DmRad9A and DmRad9B share homology over the first 268 amino acids with their human counterpart, whereas that the C-terminal region of the DmRad9 protein is unique to *Drosophila* and was not identified in the human protein.

First, to check whether the two predicated alternative splice forms are indeed transcribed, we tried to amplify them by PCR from cDNA of flies ovaries using the same forward primer and a reverse primer specific to each transcript. Both *DmRad9* alternative splice forms were amplified, demonstrating that the DmRad9 gene generates two splice variants. To determine the expression level of each of the transcripts during oogenesis, real time RT-PCR using primers specific to each transcript was performed. The real time RT-PCR data was analyzed by the 2^−ΔΔCt^ method [22]. Our results revealed that the *DmRad9A* transcript is more abundant than is the *DmRad9B* transcript (6.42±0.27-fold), suggesting that DmRad9A in the major transcribed alternative splice during oogenesis. It is worth mentioning that even though the DmRad9A transcript predominates during oogenesis, this does not necessary imply that DmRad9B plays no role during oogenesis.

### DmRad9A is also localized to the nuclear membrane in cultured mammalian cells

**Figure 2:**
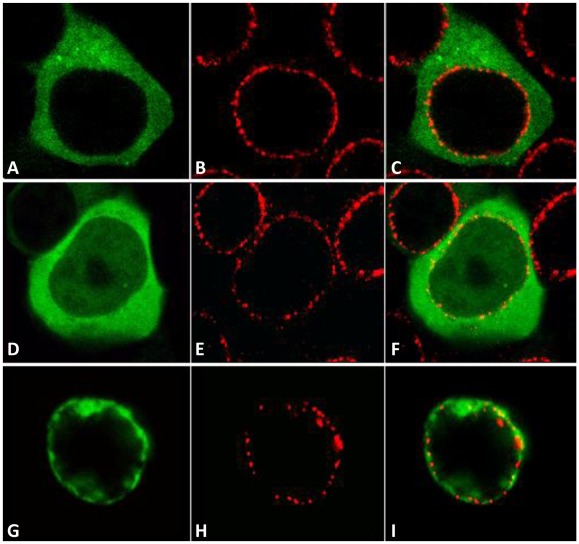
Localization of the *Drosophila* Rad9, Hus1 and Rad1 proteins in mammalian Human Embryonic Kidney 293 **(HEK293).** Confocal images of cells expressing (A) GFP-DmHus1, (D) GFP-DmRad1, and (G) GFP-DmRad9A. (B, E and H) Antibody staining of the NUP 414 protein, which recognizes several nucleoporins. (C, F and I) are merged images of (A–B), (D–E), and (G–H), respectively.

Since it was found that DmRad9A is localized to the cell nucleus membrane (Figure 1C), the localization pattern of this *Drosophila* protein in mammalian cells was determined. For that purpose, each of the 9-1-1 genes was cloned into a mammalian expression vector to yield the protein tagged to GFP. We found that similarly to their localization pattern in *Drosophila* cell line; these proteins show the same localization pattern in mammalian cells. Specifically, DmHus1 protein is localized to the cell cytoplasm (Figure 2A), DmRad1 is evenly distributed in the cell (Figure 2D) and DmRad9A is localized to the cell nucleus membrane (Figure 2G). Interestingly, an almost similar localization pattern was shown for the human homologues; both hRad1 and hHus1 are cytoplasmic while hRad9 is a nuclear protein [19]. The only difference between the Drosophila Rad9 gene and its human counterpart is that the *DmRad9* gene, but no the human gene, contains an alternative splice form that is localized to the nucleus membrane.

### DmRad9, DmRad1 and DmHus1 physically interact

**Figure 3:**
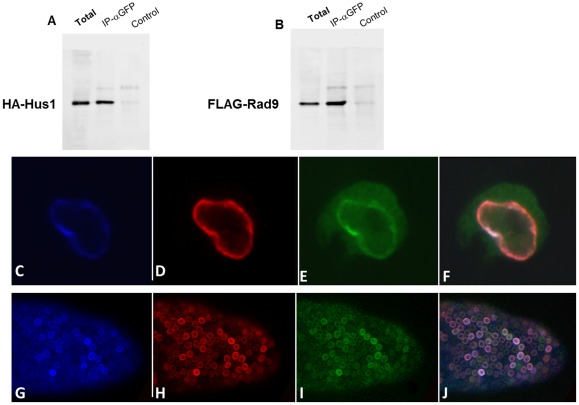
Physical interaction between DmRad9, DmRad1 and DmHus1. DmRad9 was co-expressed in S2 cells with DmRad1 and DmHus1. A total lysate of S2 cells was extracted and subjected to immunoprecipitation. (A) DmRad1 was immunoprecipitated using anti-GFP antibodies. Anti-HA antibodies were used to detect DmHus1. (B) The same blot as in (A) was probed for FLAG-DmRad9 using anti-FLAG antibodies. (C–F) Confocal images of S_2_R+ cells expressing FLAG-DmRad9, GFP-DmRad1 and HA-DmHus1. (G–J) Confocal images of follicle cells from transgenic FLAG-DmRad9::HA-DmHus1::GFP-DmRad1::*CY2Gal4* flies expressing egg chamber. (C) Staining with anti-FLAG antibodies detecting Flag-DmRad9. (D) Staining with anti-HA antibodies detecting HA-DmHus1. (E) GFP-DmRad1. (F) Merged (C–E). (G) Staining with anti-FLAG antibodies detecting Flag-DmRad9. (H) Staining with anti-HA antibodies detecting HA-DmHus1. (I) GFP-DmRad1. (J) merged G–I. Total protein served as positive control while a sample treated with protein A alone (no beads) served as negative control.

Next, the interaction between the *Drosophila* Rad9, Rad1 and Hus1 proteins was analyzed. Previously, using a yeast two-hybrid system, we reported the direct interaction of these proteins and found that Hus1 interacts with DmRad9A and Rad1, although no interaction between Rad1 and DmRad9A was detected by in this assay [20]. To more directly test whether these proteins form a complex, HA-DmHus1, FLAG-DmRad9A and GFP-DmRad1 proteins were expressed in S2 cell. Cell lysates was subjected to immunoprecipitation using anti-GFP antibodies or normal rabbit IgG as a negative control (Figure 3). Captured complexes were analyzed using antibodies specific for FLAG, HA, or GFP. Our results demonstrate for the first time that it is possible to co-precipitate DmRad9 with DmRad1 (Figure 3) and Hus1, indicating that DmRad9 forms a complex with DmRad1 and DmHus1.

### DmRad9 determines the localization of the 9-1-1 complex

It was shown that the human Rad9 protein is localized to the nucleus and that the Rad9 protein also determined the localization of the 9-1-1 complex [18]. Thus, the localization of the *Drosophila* complex when tagged versions of the three polypeptides are co-expressed, was assessed. For this purpose, transgenic flies expressing tagged versions of all three proteins were created. Expressing all three proteins in somatic follicle tissues cells lead to their accumulation at the nuclear membrane (Figure 3J), i.e. where DmRad9A was detected when expressed alone. Similar results were obtained when all three proteins were expressed in S_2_R+ cells (Figure 3F). These results confirm that DmRad9 can determine localization of the 9-1-1 complex.

### Identification of a DmRad9 nuclear localization signal

**Figure 4:**
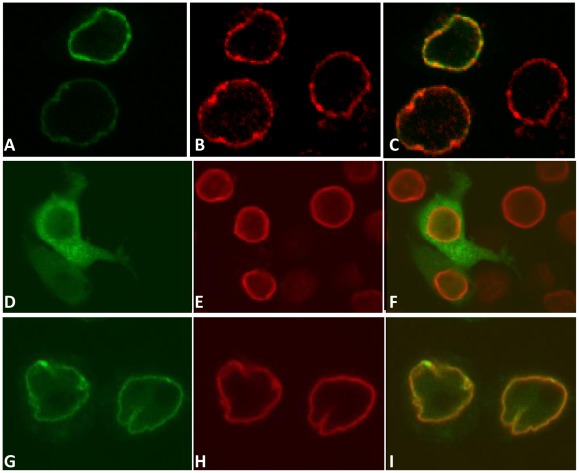
Identification of the DmRad9A nuclear localization signal. Confocal images of S_2_R+ cells expressing DmRad9A mutated in suspected NLS sequences. (A) DmRad9A mutated at position 287 – 289 (NLS1). (D) DmRad9A mutated Position 300–302 (NLS2). (G) DmRad9A mutated Position 314–316 (NLS3). (B, E and H) stained with anti-lamin antibodies, which mark the nuclear membrane, in red. (C) Merged image of (A) and (B). (F) Merged image of (D) and (E). (I) Merged image of (G) and (H).

**Figure 5:**
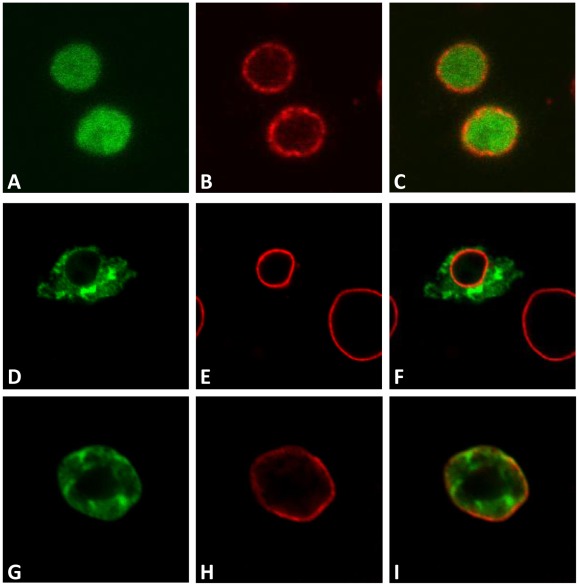
Identification of the DmRad9B nuclear localization signal. Confocal images of S_2_R+ cells expressing DmRad9A mutated in suspected NLS sequences. (A) DmRad9B mutated at position 287 – 289 (NLS1). (D) DmRad9B mutated Position 300–302 (NLS2). (G) DmRad9B mutated Position 314–316 (NLS3). (B, E and H) stained with anti-lamin antibodies, which mark the nuclear membrane, in red. (C) Merged image of (A) and (B). (F) Merged image of (D) and (E). (I) Merged image of (G) and (H).

It was shown that human Rad9 contains a predicted NLS that plays an essential role in the nuclear transport of not only Rad9 but also of human Rad1 and Hus1 [18]. As our results showed that DmRad9A is also localized to the nuclear membrane and that DmRad9B is concentrated in the nucleus, it appears that both DmRad9A and DmRa9B contain a NLS. To explore this possibility, the DmRad9A and DmRad9B proteins were scanned for NLSs using the PSORT II algorithm [23]. This search revealed three potential NLSs, the first found between amino acids 287 and 289, the second between amino acids 300 and 302 and the third between amino acids 314 and 316. To determine the importance of each potential NLS, the suspected lysine and arginine resides were mutated to alanine. We found that mutating amino acids either in DmRad9A (Figure 4) or DmRad9B (Figure 5) at sites 287–289 (Figure 4C and 5C) and 314–316 (Figure 4I and Figure 5I) to alanines had no effect on the localization of the proteins. However, mutating residues 300–302 to alanine had a dramatic effect on the localization of DmRad9A (Figure 4F) and DmRad9B (Figure 5F). In this case, the protein is no longer detected in the cell nucleus and is instead found in the cytoplasm. These results suggest that *Drosophila* DmRad9 contains a monopartite NLS between amino acids 300 and 302.

### DmRad9A localization at the oocyte nuclear membrane is not affected by over-expression of non-phosphorylatable Barrier-to-Autointegration Factor (BAF3A) but is affected by a failure to repair DSBs

To study the physiological importance of DmRad9A nuclear localization, we addressed the localization of DmRad9A is response to activation of a meiotic checkpoint. It was suggested that the interaction between DNA, protein and the nuclear membrane plays an important role during *Drosophila* meiosis [24]–[25]. It was shown that after recombination has completed, NHK-1 directly phosphorylates BAF that anchors meiotic chromosomes to the nuclear envelope [24]. Thus, mutation of NHK-1 or over-expression of non-phosphorylatable BAF (called BAF3A) resulted in an association of the chromosomes with the nuclear envelope and prevented the proper organization of the highly packed chromatin of the oocyte nucleus (also called the karyosome) [24]. BAF protein was found to link chromatin DNA to LEM-domain–containing inner nuclear envelope proteins (i.e. *Drosophila* otefin) by binding to both simultaneously. In cells expressing non-phosphorylatable BAF-3A, otefin often accumulated in a region of the nuclear envelope in close contact with meiotic chromosomes [24].

**Figure 6:**
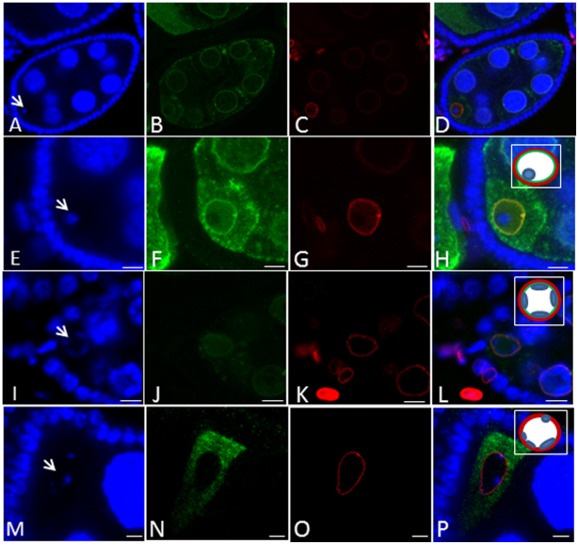
Effects of meiotic checkpoint activation on DmRad9A oocyte nuclear membrane localization. Confocal images of stage 7 egg chambers. (A, E, I and M) are stained for DNA (blue, arrows mark oocyte nucleus DNA, karyosome); arrows mark the oocyte nucleus (karyosome). (B, F, J and N) GFP-DmRad9A (green). (C, G, K and O) are stained with anti-lamin antibodies, which mark the nuclear membrane, in red. (Inset in H, L and P), represents a schematic description of the oocyte nucleus. Red-lamin, green-GFP-DmRad9A and blue-karyosome. (A–H) GFP-DmRad9A:: *nosGal 4-VP16* egg chamber, E–H are enlargement of the oocyte region from A–D, respectively. (I-L) BAF3A:: GFP-DmRad9A:: *nosGal 4-VP16* egg chamber. M–P, GFP-DmRad9A:: *nosGal 4-VP16; okr^AA^*/*okr^RU^*.

Taken into account that DmHus1, as part of the 9-1-1 complex, is involved in the meiotic checkpoint pathway, together with the fact that DmRad9A is localized to the nuclear membrane, we determined if DmRad9A localization is affected by over-expression of BAF3A. We first found that expression of GFP-DmRad9A in the germline under the control of nos Gal4-VP16 led to the accumulation of the protein at the nuclear membrane in both nurse cells and in the developing oocyte (Figure 6B). Next, BAF3A was over-expressed in the ovaries and the localization of GFP-DmRad9A was analyzed. We found that affecting karyosome formation ([24]; Figure 6I) by over-expression of BAF3A had no effect on the localization of DmRad9, with the protein remaining at the oocyte nuclear envelope (Figure 6J).

Finally, we asked whether activation of the meiotic checkpoint in response to the presence of unrepaired DSBs would affect DmRad9A localization. Accordingly, the localization of DmRad9A in a DNA repair mutant background was studied. Thus, flies that are homozygous for *okra (okr)*, the *Drosophila* Rad54 homologue, and that express GFP-DmRad9 under the control of nos Gal4-VP16 were generated. We found, as described before, that the karyosome of the *okr* mutant is fragmented ([26]; Figure 6M). Moreover, GFP-DmRad9 was no longer located at the nuclear membrane (Figure 6N), suggesting that persistence of DSBs during meiosis affects DmRad9A localization.

## Discussion

In this study, we showed that the *Drosophila* 9-1-1 proteins present an almost similar localization pattern as do their mammalian homologues. Both hRad1 and hHus1 are cytoplasmic, while hRad9 is a nuclear protein [19]. In *Drosophila* the two alternatively spliced forms of DmRad9 are both localized to the nucleus, yet DmRad9A (but not the human homologue) is a nuclear membrane-bound protein. Moreover, when all three proteins were co-over-expressed, DmRad9A and DmRad9B determined the localization of the two other proteins, namely DmRad1 and DmHus1. The same is also true in mammalian cells, where it was suggested that genotoxic stress induces the expression of hRad1, which in turn stabilizes hHus1. Once all proteins are present, hRad9 transports the complex into the nucleus [19].

We have shown that DmRad9 possesses a NLS near the C-terminus of the protein. Much like its human counterpart [18], the DmRad9 NLS was found to be crucial for localization of the protein to the nucleus. Interestingly, the NLS of hRad9 and DmRad9 reside in the C-terminal regions of both proteins, no other similarity exists between these two proteins in this region. The first 274 amino acids of the human Rad9 protein show relatively high similarity (52%) to the first 268 amino acids of DmRad9. Moreover, both the human Rad9 NLS motif, which lies between amino acids 356 to 364, and the DmRad9 NLS motif, found between amino acids 300 to 302, are not conserved. Despite these differences, DmRad9A is localized to nuclear membrane when expressed in mammalian cells, suggesting that the mechanism by which the protein is targeted to the nuclear membrane is likely conserved.

Previously, it had been shown that *DmHus1* is involved in activation of a meiotic checkpoint [20]. Moreover, as described in this study, the *DmRad9A* transcript is more abundant than is the *DmRad9B* transcript during oogenesis. Thus, the physiological function of DmRad9A nuclear membrane localization during activation of the meiotic checkpoint was studied. The *Drosophila* meiotic checkpoint was first revealed upon study of a class of mutant genes that required for the repair of recombination-induced DSBs during *Drosophila* oogenesis [26]–[30]. Mutations in these genes lead to activation of a meiotic checkpoint [28], [31]–[32], leading to the appearance of several defects during oogenesis. The most obvious phenotypes manifested are the dorsal-ventral patterning defects of the egg and the organization of the oocyte nucleus karyosome. Recent studies have offered some insight into the connection between activation of the meiotic checkpoint and the karyosome. It was found that nucleosomal histone kinase-1 (NHK-1) is essential for karyosome formation [33]. NHK-1 phosphorylates the linker, BAF, to release meiotic chromosomes from the oocyte nuclear envelope during karyosome formation [24]. Expression of a non-phosphorylatable BAF3A mutant prevented the release of meiotic chromosomes and resulted in a karyosome defect, as was observed in NHK-1 mutants [25]. Based on the above, we decided to analyze whether DmRad9A oocyte nuclear localization could be affected in response to an inability to repair DSBs or due to failure in releasing meiotic chromosomes from the oocyte nuclear envelope. For this purpose, we considered the localization of GFP-tagged DmRad9A in *okr* mutants, a Rad54-like protein, a double-strand DNA breaks repair enzyme [26], and in flies expressing a non-phosphorylatable form of BAF (BAF3A). DmRad9A localization is not affected in the background of over-expression of BAF3A. Were DmRad9A involved in the physical connection between the chromosomes and the oocyte nuclear envelope, we would have expected to get results similar to what was shown for otefin. In the wild type oocyte nucleus, otefin is found at the nuclear membrane. However, upon over-expression of BAF3A, otefin accumulated in a region of the nuclear envelope in close contact with meiotic chromosomes and was absent from other region of the oocyte nuclear membrane [24]. On the other hand, persistence of DSBs, as observed in *okr* mutants, dramatically affected DmRad9A oocyte nuclear membrane localization. Thus, the displacement of DmRad9A from the oocyte nuclear membrane due to activation of a meiotic checkpoint is probably part of the oocyte response to DSBs, rather than reflecting a step in the process of attachment of the meiotic chromosome to the nuclear membrane.

## Materials and Methods

### Fly strains

Flies were cultured in standard cornmeal/agar medium at 25°C. The Oregon-R and relevant Gal4 driver strains were used as wild type controls. The following mutant and transgenic flies were used: *okr^AA^*, *okr^RU^* [26], pUASp-HA-Hus1 [20], pUASp-Flag-Rad9A [30] and pUASp.

BAF3A [24]. Germline and follicle cell expression was performed with P{GAL4::VP16-nos.UTR}CG6325^MVD1^, subsequently referred to as nos Gal4-VP16 [34] and *CY2*-Gal4 in each case [35], respectively.

### Cloning of *rad1* and *rad9* into pUASp vectors and creation of transgenic flies

To create GFP-tagged DmRad1, *DmRad1* was amplified from cDNA using modified primers to create an XbaI restriction site at the 5' end (5' TCTAGAATGACTGATGTGGA GCCATCGCCC 3'), and a NotI restriction site at the 3' end (5'GCGGCCGCTTAATCAGTGTGAG TGTGAGCAAAGGAATTATG 3'). The resulting PCR product was digested with Xba and NotI and cloned into the pUASp vector containing GFP. To create GFP-tagged DmRad9, *DmRad9* was amplified from cDNA using modified primers to create an XbaI restriction site at the 5' end (5' TCTAGAGTTTGTTTACAAATTTCAGC3'), and an XbaI restriction site at the 3' end (5'TCTAGATGCTTTTAAAATTTATGTTT 3'). The resulting PCR product was digested with XbaI and cloned into the pUASp vector containing GFP. P-element-mediated germline transfection of these constructs was carried out according to standard protocols [36].

### Cloning of *rad9*, *rad1* and *hus1* in a mammalian vector

9-1-1 proteins were cloned into the pEGFP-C3 vector (28). The full-length coding sequences of all 9-1-1 proteins were amplified from cDNA by PCR. The *DmRad9A* sequence was amplified using modified primers to create an XhoI restriction site at the 5' end (5' CTCGAGATGAAATACACTTTAGAGGG 3') and a KpnI site at the 3' end (5' GGTACCTCA AAGCAGCTCGTAACC 3') of the gene. The full-length DNA sequence of *DmHus1* was amplified by PCR using modified primers to create an XhoI restriction site at the 5' end (5' CTCG AGATGAAGTTCCGCGCA CTGATGC 3') and a KpnI site at the 3' end (5' GGTACCCTACATACAAAC AGCTGGC 3') of the gene. To express DmRad1, the coding sequence was amplified using modified primers to introduce a HindIII restriction site at the 5' end (5' AAGCTTATGACTGATGTGGAGCCATCGC 3') and a KpnI site at the 3' end (5' GG TACCTTAATCAGTGTTGAGCAAAGG 3') of the gene. The resulting *DmRad9A* and *DmHus1* PCR products were digested with XhoI and KpnI and cloned into the pEGFP-C3 vector. *DmRad1* was digested with HindIII and KpnI and cloned into the pEGFP-C3 vector.

### Real-time reverse-transcription (RT)-PCR

Total RNA was extracted from ovaries using the NucleoSpin RNA II kit, including DNase treatment, according to the manufacturer's instructions (Macherey-Nagel). cDNA was transcribed from 1–5 μg total RNA using reverse transcriptase and oligo(dT) (Bio-Lab, Beit Haemek, Israel), again according to the manufacturer's instructions (ABgene). Reverse-transcribed total RNA (100 ng) was amplified in a 20 μl reaction containing 100 nM of each primer and 10 μl of SYBR Green PCR Master Mix (Stratagene). RT-PCR was performed to amplify *DmRad9A* and *DmRad9B* cDNA using the same forward primer (5'-GCACGGAGGTTTGCTTTATC-3') and the *DmRad9A* (5'-CAACATAGTCTTCAGTCGGC-3') or *DmRad9B* (5'- GTAGGTCCTCTGAAAGCAAC -3') reverse primers. To normalize differences in total cDNA between samples, ribosomal protein 49 cDNA was amplified using primers Rp49 Fwd (5'-CCGCTTCAAGGGACAGTATCTG-3') and Rp49 Rev (5'-CACGTTGTGCACCAGGAACTT-3'). PCR conditions were as follows: The reaction mixtures were first kept at 95°C for 15 min, then 40 cycles of PCR (95°C for 30 s, 55°C for 1 min, and 72°C for 1 min) were performed, and finally, the mixtures were incubated at 95°C for 1 min, 55°C for 30 s, and 95°C for 30 s. All quantitative PCR analyses were performed in triplicate. Real-time PCR was performed using the Mx3000p cycler (Stratagene, La Jolla, CA) and the amount of gene product in each sample was determined by the comparative quantification method, using MxPro software (Stratagene).

### Co-immunoprecipitation assay

Cells expressing 9-1-1 constructs were treated with lysis buffer (PBS, 1% Triton X-100 and protease inhibitors). Pre-cleared extracts were incubated overnight at 4°C with anti-GFP rabbit antibodies (1∶250, Sigma). Immuno-complexes were recovered by incubation with protein A-coated beads (Adar Biotech) for 2 h at 4°C. To detect interactions between proteins, western blotting with anti-HA mouse monoclonal antibodies (1∶1000; Santa Cruz) or anti-Flag mouse monoclonal antibodies (1∶1000; Sigma) antibodies was performed. As a negative control, cell lysate was precipitated with normal rabbit IgG (1∶250, Santa Cruz Biotechnology).

### Transformation of Human Embryonic Kidney 293 (HEK293) cells

T-REx-293 cells (HEK cells stably containing the pcDNA6/TR regulatory vector and thus expressing the tetracycline repressor) (Invitrogen) were cultured in DMEM medium (Gibco) enriched with glucose, 10% fetal calf serum (FCS), 2 mM L-glutamine and a 1% antibiotic mixture comprising penicillin and streptomycin (Biological Industries). Cells were then incubated at 37°C and 92% humidity in the presence of 5% CO_2_. 5×10^6^ cells were transfected with 1 µg of the expression vector and 1 µg of a plasmid containing the actin-Gal4 driver using the TransIT-LT1 reagent (Mirus), according to the manufacturer's protocol. Prior to transfection, the growth medium was replaced with fresh DMEM. 24 h post-transfection, the medium was again replaced.

### Cell fixation and staining

S_2_ or S_2_R+ cells were cultured in *Drosophila* Schneider's medium (Biolabs Industries, Israel) containing 10% fetal calf serum and PSA solution containing penicillin (10,000 U/ml), streptomycin (10 mg/ml) and amphotericin B (0.025 mg/ml) (1:100;, Biolabs Industries). Cells were maintained at 25°C under normal atmospheric conditions. Prior to transfection, the cells were cultured in fresh Schneider's medium. 4×10^6^ cells were transfected with 1 μg of tej pUASp-based expression vector and the Actin-Gal4 driver using Escort IV (Sigma), according to the manufacturer's protocol. 24 h post-transfection, the medium was replaced.

Cells were fixed for 15 min with 3.8% formalin in PBS, washed 3×1 min in PBS and then incubated for 4 min in PBS containing 0.3% Triton X-100 (PBST). Samples were then incubated for 1 h with primary antibodies at appropriate dilutions with 0.2% fish skin gelatin (FSG). After a PBS wash, the cells were incubated for 1 h with secondary antibodies, again at appropriate dilutions and with 0.2% FSG. After another wash with PBS, the cells were mounted in 50% glycerol and imaged using a confocal microscope. The primary antibodies used were rat α- HA (1∶250; Sigma), rabbit α– HA (1∶250; Santa Cruz), mouse α-Flag M2 (1∶250; Sigma), mouse α-Lamin (1∶50; Hybridoma Bank, Iowa University), and mouse α-Nup 414 (1∶3000). As secondary antibodies, we used Cy2 goat α-rabbit (1∶500; Molecular Probes), Cy2 goat α- mouse (1∶100; Jackson Immunoresearch), Cy3 goat α-mouse (1∶100; Jackson Immunoresearch), Cy3 goat α-rat (1∶100; Jackson Immunoresearch), Cy3 goat α-rabbit (1∶100; Jackson Immunoresearch), and Cy5 goat α-mouse (1∶100; Jackson Immunoresearch) antibodies. DNA was stained using DAPI (1∶1000).

### Western blot analysis

Proteins were loaded onto a 10% polyacrylamide gel. Following electrophoresis, proteins were transferred to nitrocellulose membranes (PROTRAN, Schleicher & Schuell) for 1 h at 300 mA. The nitrocellulose membranes were blocked by incubation in TTBS (0.2 M Tris, pH? 1.5 M NaCl, 9 mM Tween 20) containing 5% non-fat dry milk for 30 min at room temperature, followed by a 1 h incubation with primary antibodies. The membranes were washed in TTBS and incubated for 30 min with labeled α-mouse antibodies (Amersham) at a 1∶2000 dilution. Antibody binding was visualized using an enhanced chemiluminesence detection kit (Biological Industries). Primary antibodies used were mouse α-HA (1∶500; Santa Cruz Biotechnology), mouse α-GFP (1∶1000; Roche Diagnostics) and mouse α-tubulin (1∶1000; Sigma) antibodies.

### Ovary antibody-staining

Ovaries were dissected in PBS, fixed for 20 min in 3.8% formaldehyde in PBS and heptane and washed 3×10-min in PBST. The ovaries were incubated for 1 h in PBS, 1% Triton X-100, and blocked for 1 h in 3% BSA in PBST. After overnight incubation at 4°C with primary antibodies at appropriate dilutions followed by PBST washes, the ovaries were incubated with secondary antibodies for 1 h, washed, and mounted in 50% glycerol. As primary antibodies, we used mouse α- Flag M2 (1∶250; Sigma) and mouse α- Flag M2 (1∶250; Sigma) antibodies. The secondary antibodies, Cy3 goat α-mouse antibodies (Jackson Immunoresearch), were used at a 1∶100 dilution. Egg chambers were imaged on a Zeiss LSM510 laser-scanning confocal microscope.

## References

[1] Sancar A, Lindsey-Boltz LA, Ünsal-Kaçmaz K, Linn S (2004). Molecular mechanisms of mammalian DNA repair and the DNA damage checkpoints.. Annu Rev Biochem.

[2] Gembka A, Toueille M, Smirnova E, Poltz R, Ferrari E (2007). The checkpoint clamp, Rad9-Rad1-Hus1 complex, preferentially stimulates the activity of apurinic/apyrimidinic endonuclease 1 and DNA polymerase β in long patch base excision repair.. Nucleic Acids Res.

[3] Guan X, Madabushi A, Chang DY, Fitzgerald ME, Shi G (2007). The human checkpoint sensor Rad9-Rad1-Hus1 interacts with and stimulates DNA repair enzyme TDG glycosylase.. Nucleic Acids Res.

[4] Parrilla-Castellar ER, Sonnet JH, Arlander SJH, Karnitz L (2004). Dial 9-1-1 for DNA damage: the Rad9-Hus1-Rad1 (9-1-1) clamp complex.. DNA Repair.

[5] Barbour L, Ball LG, Zhang K, Xiao W (2006). DNA Damage Checkpoints Are Involved in Postreplication Repair.. Genetics.

[6] Aylon Y, Kupiec M (2003). The checkpoint protein Rad24 of *Saccharomyces cerevisiae* is involved in processing double-strand break ends and in recombination partner choice.. Mol Cell Biol.

[7] Grushcow JM, Holzen TM, Park KJ, Weinert T, Lichten M (1999). Saccharomyces cerevisiae checkpoint genes MEC1, RAD17 and RAD24 are required for normal meiotic recombination partner choice.. Genetics.

[8] Pandita, RK, Sharma GG, Laszlo A, Hopkins KM, Davey S (2006). Mammalian rad9 plays a role in telomere stability, S- and G2-Phase-specific cell survival, and homologous recombinational repair.. Mol Cell Biol.

[9] Komatsu K, Miyashita T, Hang H (2000). Human homologue of S. pombe Rad9 interacts with BCL-2/BCL-xL and promotes apoptosis.. Nat Cell Biol.

[10] Lee MW, Hirai I, Wang HG (2003). Caspase-3-mediated cleavage of Rad9 during apoptosis.. Oncogene.

[11] Yoshida K, Komatsu K, Wang HG, Kufe D (2002). c-Abl Tyrosine Kinase Regulates the Human Rad9 Checkpoint Protein in Response to DNA Damage.. Mol Cell Biol.

[12] Yin Y, Zhu A, Jin YJ, Liu YX, Zhang X (2004). Human RAD9 checkpoint control/proapoptotic protein can activate transcription of p21.. PNAS.

[13] Lieberman HB (2006). Rad9, an Evolutionarily Conserved Gene With Multiple Functions for Preserving Genomic Integrity.. J Cell Biochem.

[14] Venclovas C, Thelen MP (2000). Structure-based predictions of Rad1, Rad9, Hus1 and Rad17 participation in sliding clamp and clamp-loading complexes.. Nucleic Acids Res.

[15] Doré AS, Kilkenny ML, Rzechorzek NJ, Pearl LH (2009). Crystal structure of the rad9-rad1-hus1 DNA damage checkpoint complex – implications for clamp loading and regulation.. Mol Cell.

[16] Sohn SY, Cho Y (2009). Crystal structure of the human rad9-hus1-rad1 clamp.. J. Mol Biol.

[17] Xu M, Bai L, Gong Y, Xie W, Hang H (2009). Structure and functional implications of the human Rad9-Hus1-Rad1 cell cycle checkpoint complex.. J Biol Chem.

[18] Hirai I, Wang HG (2002). A Role of the C-terminal Region of Human Rad9 (hRad9) in Nuclear Transport of the hRad9 Checkpoint Complex.. J. Biol Chem.

[19] Hirai I, Sasak, T, Wang HG (2004). Human hRad1 but not hRad9 protects hHus1 from ubiquitin-proteasomal degradation.. Oncogene.

[20] Abdu U, Klovstad M, Butin-Israeli V, Bakhrat A, Schüpbach T (2007). An essential role for Drosophila hus1 in somatic and meiotic DNA damage responses.. J. Cell Science.

[21] Peretz G, Gur-Arie L, Bakhrat A, Abdu U (2009). The *Drosophila hus1* gene is required for homologous recombination repair during meiosis.. Mech Dev.

[22] Livak KJ, Schmittgen TD (2001). Analysis of relative gene expression data using real- time quantitative PCR and the 2^-ΔΔ*CT*^ method.. Methods.

[23] Nakai K, Kanehisa M (1992). A knowledge base for predicting protein localization sites in eukaryotic cells.. Genomics.

[24] Lancaster OM, Cullen CF, Ohkura H (2007). NHK-1 phosphorylates BAF to allow karyosome formation in the Drosophila oocyte nucleus.. J Cell Biol.

[25] Lancaster OM, Breuer M, Cullen CF, Ito T, Ohkura H (2010). The meiotic recombination checkpoint suppresses NHK-1 kinase to prevent reorganisation of the oocyte nucleus in Drosophila.. PLoS Genet.

[26] Ghabrial A, Ray RP, Schüpbach T (1998). okra and spindle-B encode components of the RAD52 DNA repair pathway and affect meiosis and patterning in Drosophila oogenesis.. Genes Dev.

[27] Abdu U, Gonzalez-Reyes A, Ghabrial A, Schüpbach T (2003). The Drosophila spn-D gene encodes a RAD51C-like protein that is required exclusively during meiosis.. Genetics.

[28] Staeva-Vieira E, Yoo S, Lehmann R (2003). An essential role of DmRad51/SpnA in DNA repair and meiotic checkpoint control.. EMBO.

[29] McCaffrey R, St Johnston D, González-Reyes A (2006). Drosophila mus301/spindle-C encodes a helicase with an essential role in double-strand DNA break repair and meiotic progression, Genetics.

[30] Klovstad M, Abdu U, Schüpbach T (2008). Drosophila *brca2* Is Required for Mitotic and Meiotic DNA Repair and Efficient Activation of the Meiotic Recombination Checkpoint.. PLoS Genet.

[31] Ghabrial A, Schüpbach T (1999). Activation of a meiotic checkpoint regulates translation of Gurken during Drosophila oogenesis.. Nat Cell Biol.

[32] Abdu U, Brodsky M, Schüpbach T (2002). Activation of a meiotic checkpoint during Drosophila oogenesis regulates the translation of Gurken through Chk2/Mnk.. Curr Biol.

[33] Ivanovska I, Khandan T, Ito T, Orr-Weaver, TL (2005). A histone code in meiosis: the histone kinase, NHK-1, is required for proper chromosomal architecture in Drosophila oocytes.. Genes Dev.

[34] Van Doren M, Williamson AL, Lehmann R (1998). Regulation of zygotic gene expression in Drosophila primordial germ cells.. Curr Biol.

[35] Queenan AM, Barcelo G, Van Buskirk C, Schüpbach T (1999). The transmembrane region of Gurken is not required for biological activity, but is necessary for transport to the oocyte membrane in Drosophila.. Mech Dev.

[36] Spradling AC, Rubin GM (1982). Transposition of cloned P elements into Drosophila germ line chromosomes.. Science.

